# Ecosystem Protection through Myco-Remediation of Chromium and Arsenic

**DOI:** 10.3390/jox13010013

**Published:** 2023-03-09

**Authors:** Neel Kamal, Jagdish Parshad, Baljeet Singh Saharan, Monika Kayasth, Vishal Mudgal, Joginder Singh Duhan, Balwan Singh Mandal, Pardeep Kumar Sadh

**Affiliations:** 1 Department of Microbiology, Chaudhary Charan Singh Haryana Agricultural University, Hisar 125004, India; 2 Central Institute for Research on Buffaloes, Hisar 125001, India; 3 Department of Biotechnology, Ch. Devi Lal University, Sirsa 125055, India; 4 Department of Forestry, Chaudhary Charan Singh Haryana Agricultural University, Hisar 125004, India

**Keywords:** arsenic, bioremediation, chromium, fungal isolates, minimum inhibitory concentration, agro-ecology, natural farming

## Abstract

The current study emphasizes fungi as an important tool against heavy metals and how isolated fungal species can be used to create a successful strategy for the bioremediation of chromium and arsenic-contaminated sites/soils. Globally, heavy metal pollution is a serious issue. In the current investigation, contaminated sites were chosen, and samples could be taken from various localities of Hisar (29.1492° N, 75.7217° E) and Panipat (29.3909° N, 76.9635° E), India. A total of 19 fungal isolates were obtained from the collected samples through the enrichment culture technique using PDA media supplemented with Cr as chromic chloride hexahydrate (50 mg/L) and As as sodium arsenate (10 mg/L) and the potential of fungal isolates to be used for the removal of heavy metals was examined. The isolates were screened for minimum inhibitory concentrations (MIC) exhibiting tolerance capabilities, and the four best isolates C1, C3, A2, and A6 with the highest MICs (>5000 mg/L), were chosen for further investigations. To use the chosen isolates in the remediation of heavy metals (Cr and As), the culture conditions were optimized. The fungal isolates C1 and C3 estimated the highest removal of 58.60% and 57.00% at 50 mg/L chromium concentration, while the isolates A6 and A2 recorded the highest removal efficiency of 80% and 56% at 10 mg/L arsenic concentration under optimal conditions. Finally, the chosen fungal isolates C1 and A6 were molecularly identified as *Aspergillus tamarii* and *Aspergillus ustus*, respectively.

## 1. Introduction

Environment protection has become one of our prime concerns in prevailing conditions. Increased industrialization and urbanization and even specific repair sites have raised the issue of heavy metal pollution in the environment. Heavy metals having a specific density of more than 5 g/cm^3^ are considered hazardous pollutants globally [1]. High toxicity, non-biodegradability, and the subsequent build-up of heavy metals in the environment make the problem more severe. Industrial effluents and municipal wastes are either discharged into water bodies or directly supplied to the fields [2]. This results in serious health issues in humans due to the accumulation of heavy metals in the human body [3]. Among various heavy metals, Arsenic (As) and Chromium (Cr) contamination has become a major problem. Chromium holds the first rank among carcinogenic substances [4]. Based on oxidation, chromium usually occurs in two forms: Cr (III) and Cr (VI). The former is less toxic and mobile than the latter. However, Cr (VI) reacts with other particles that are present in the air and changes to Cr (III), which is more stable than Cr (VI) [5,6,7]. There are various sources of chromium emissions in the environment, including anthropogenic sources, which include tanneries, steel industries, and fly ash [8]. Chromium toxicity leads to various health issues, including fatal chronic diseases [9]. The presence of arsenic in the groundwater and soil of many developing countries such as India, Thailand, Bangladesh, Nepal, Argentina, and Poland is a major health alarm [10]. Inorganic forms which are mainly present in the environment are Arsenate (V) and Arsenite (III). Inorganic forms of arsenic are more toxic compared to organic ones and are inter-convertible [11]. The pentavalent form of arsenic [As (V)] is a structural analog of inorganic phosphate and substitute phosphate in mitochondrial pathways and glycolysis [12]. The major sources of arsenic contamination are more natural than anthropogenic [13,14,15,16,17]. The tolerance limit of arsenic in drinking water was 10 μg/L as per World Health Organization guidelines, and it was mainly deposited in the nails, hairs, bones, and vital organs, such as the liver and kidneys [18,19]. There are several conventional methods for the elimination of heavy metals, which include chemical precipitation, ion exchange, ultra-filtration, reverse osmosis, electro-winning, carbon adsorption, and solvent extraction. The majority of these are expensive and unfit due to the release of highly hazardous toxic pollutants as by-products. Their high cost and environmental concerns due to the production of toxic by-products make them less effective [20,21]. Many bacteria can bioremediate in wastewaters, especially in the distillery [22] and textile [23] effluents. The bacterial cultures remediate these effluents as individuals or as a consortium [24] for various enzymes produced by microbes which can also be utilized for this purpose [25]. The plant growth-promoting rhizobacteria (PGPR) can remediate the contaminated sites as they are tolerant to a certain level of heavy metals [26,27]. Plants have the potential, through various molecular and physiological mechanisms, to alleviate abiotic stresses [28]. Plant–microbial interactions can play a very important role in such remediation processes [29,30]. Thus, the need of the hour is to choose eco-friendly approaches to deal with elevated levels of heavy metals in the environment.

Mycoremediation is one of the most promising and eco-friendly approaches to the bioremediation of chromium and arsenic. Fungi have been known for their ability to adapt to harsh environmental conditions, such as pH, nutrient availability, temperature, and high metal concentrations. Fungi secrete various enzymes throughout their life cycle, which also helps in the bioremediation of metals. Fungal cell walls are constituted of various groups such as polysaccharides and proteins with carboxyl, sulfate, amino, hydroxyl, and phosphate groups for the binding of metal ions and which act as the most effective biosorbent for the removal of heavy metals [31,32,33]. Until now, several fungal species have been identified as *Aspergillus flavus*, Aspergillus fumigates, Fusarium proliferatum, Penicillium radicum, Beauvariabassiana, etc., for the bioremediation of these heavy metals [34,35,36,37,38,39]. Many fungal strains have been observed for the removal of heavy metals such as *Phanerochaete chrysosporium*, *Aspergillus awamori*, *Aspergillus flavus*, and *Trichoderma viride* from heavy metal-contaminated wastewater and industrial effluents [40]. In view of the above problem, the present investigation was taken to investigate the ability of indigenous fungal species to deal with selected heavy metals and to study the efficiency of heavy metal removal from the liquid medium. In short, a heavy metal uptake by fungal isolates was determined.

## 2. Materials and Methods

### 2.1. Sample Collection

Soil samples were collected from eleven regions of the Hisar and Panipat districts of Haryana, India. Soil samples were collected from auto-market areas, steel and textile industries, and farmer’s fields receiving sewage water. The collected soil samples were stored in a refrigerator at 4 °C in polyethylene bags until their utilization.

### 2.2. Estimation of Physicochemical Properties of Soil Samples

#### 2.2.1. Physical Properties

The texture of the soil was determined qualitatively by a rapid feel method by rubbing the moistened soil between the thumb and fingers.

#### 2.2.2. Chemical Properties

- The pH of the samples was estimated using a digital pH meter.
- The digital conductivity meter was used to estimate the electrical conductivity of the samples.
- Carbon content in the samples was determined by the wet-digestion method [41].
- The total nitrogen content was determined by Kjeldahl’s method [42].
- The method that [43] used was to determine the total phosphorus of the samples.
- The total potassium of the samples was estimated on a flame photometer by direct feeding.
- The total chromium and arsenic levels in soil samples were determined by the method described by McGrath and Cunliffe [44].

### 2.3. Isolation of Fungal Isolates and Their Maintenance

The isolation was carried out using the enrichment culture technique. Ten grams of soil were taken in a 90 mL potato dextrose broth amended with chromium as chromic chloride hexahydrate (50 mg/L) and arsenic as sodium arsenate (10 mg/L). The flasks containing broth were incubated at 30 °C on the shaker for seven days. Ten ml of the sample from these flasks was transferred to new flasks containing fresh potato dextrose broth. The freshly cultured flasks were incubated at 30 °C on the shaker. After seven days, the enriched samples were diluted and plated on PDA media plates containing Cr (III) (50 mg/L) and As (V) (10 mg/L). The plates were incubated at 30 °C for five days. The different colonies of fungi were picked and purified to obtain pure cultures and were maintained on potato dextrose agar slants.

### 2.4. Screening of Fungal Isolates

The purified fungal isolates were screened and selected based on Cr and As tolerance. The tolerance of both metals was determined in terms of their minimum inhibitory concentration (MIC). All the fungal isolates were streaked on PDA plates amended with chromium (100–5000 mg/L) and arsenic (100–5000 mg/L). Streaking on PDA plates without metal served as the control. The PDA plates were incubated for five days at 30 °C for growth. The highest concentration at which no visible growth of the fungi occurred was assumed to be the minimum inhibitory concentration (MIC).

### 2.5. Characterization of Fungal Isolates

The selected fungal isolates showing high MIC were examined morphologically and biochemically. Lacto-phenol cotton blue staining was performed on a 3–4 days old culture of fungus and examined microscopically (40×) to observe fungal hyphae and spores [45]. The isolates were also characterized biochemically for various tests such as the carbohydrate assimilation test [46], cellulose [47], laccase [48], amylase [49], and pectinase test [50].

### 2.6. Optimization of Cultural Conditions

The culture conditions were optimized to support the maximum growth of isolated fungi so that they could be further used for the biosorption of metals from the liquid medium. The effect of a wide range of pH (2–7) on the growth of selected fungal isolates was studied by adjusting the pH with 1 N HCl and 1 N NaOH while the other conditions were kept constant. The spore suspension was prepared and transferred into a potato dextrose broth (100 mL) with a different pH over a range of 2–7 which was then incubated at 30 °C for five days. After five days of incubation, the media was centrifuged, and the wet weight of the pellet was analyzed. Similarly, the other parameters, such as the incubation temperature (25 °C, 30 °C, 35 °C, and 40 °C) and carbon sources (galactose, dextrose, fructose, and sucrose) were studied.

### 2.7. Heavy Metals Removal Efficacy

The selected isolates were inoculated in a modified potato dextrose medium containing sucrose as the carbon source instead of dextrose. The medium containing 50 mg/L, 100 mg/L, and 200 mg/L of chromium and 10 mg/L, 50 mg/L, and 100 mg/L of arsenic were suspended separately into flasks and sterilized. The pH of the medium was adjusted to five. The flasks with the broth were inoculated with one ml spore suspension from a seven-day-old slant culture and incubated at 30 °C for 5–6 days. Un-inoculated flasks containing heavy metals served as a control. The fungal biomass was harvested after 96 h using Whatman filter paper. The reduction in heavy metal concentrations in the filtrate (filtered broth) was estimated using AAS (Atomic Absorption Spectroscopy) with an air–acetylene flame. The following equation determined the percent metal removal [51].

% Removal = [(Co − C)/Co] × 100(1)

where Co = the initial concentration of the metal (mg/L)

C = the final concentration of the metal after biosorption (mg/L)

### 2.8. Molecular Characterization of Fungal Isolates

The fungal isolates C1 and A6 were characterized molecularly by Biologia Research India Private Limited, New Delhi. The ITS DNA region of isolates C1 and A6 for about a 650 bp amplicon was amplified using Universal primers:

ITS1: TCCGTAGGTGAACCTGCGG

ITS4: TCCTCCGCTTATTGATATGC

A single PCR sample containing 1μL of DNA was amplified in a 20 μL reaction mixture containing a 10× Buffer, each deoxynucleoside triphosphate at 0.2 mM, each primer at 0.5 μM, and 2 U of Taq polymerase (R001C TaKaRa Taq™) per ml. PCRs were subjected to Initial denaturation for 3 min at 95 °C, 32 cycles (denaturation, 30 s at 95 °C; annealing, 30 s at 50 °C; extension, 1 min at 72 °C) and 1 final extension cycle at 72 °C for 10 min. Twenty microliters of the reaction mixtures were analyzed on 1.5% agarose/Tris-Cl-sodium acetate-EDTA in the presence of 0.5 μg of ethidium bromide per ml and photographed under UV illumination. The positive amplicons were purified through the use of a FavorPrep™ GEL/PCR Purification Kit (Cat No. FAGCK 001). The Purified Product was sequenced with Sanger’s method of DNA sequencing for both directions. The positive amplicons were purified by using a FavorPrep™ GEL/PCR Purification Kit (Cat No. FAGCK 001). The purified product was sequenced with Sanger’s method of DNA sequencing for both directions. The sequence data were compared with the data stored in NCBI (National Centre for Biotechnology Information) through BLAST (Basic Local Alignment Search Tool). The phylogenetic tree was constructed by MEGA 11 software using the neighboring Clustal W method.

## 3. Results and Discussion

### 3.1. Sample Collection and Analysis of Physico-Chemical Properties

Samples of heavy metal-contaminated soil were collected from different locations in Haryana, and physicochemical analyses were performed. The electrical conductivity of the samples showed a huge variation from 0.51 to 2.50 mS/cm (Table 1). The pH values of the samples did not show much variation. The organic carbon ranged from 0.16% to 0.68%. The organic carbon content was observed at a maximum at site no. 5 due to the high carbon content of sewage waste. It determines the soil’s ability to hold and immobilize heavy metals. The amount of nitrogen, phosphorus, and potassium was highest at site no. 3, 5, 10, and 11, among other sites, due to the high content of organic, inorganic, and nitrogenous compounds in sewage, textile, and tannery wastewater. Similar results were determined by Angin et al. [52]. High levels of chromium were found at sites no. 10 (1.80 mg/L) and site no. 11 (2.23 mg/L) due to high chromium in the textile and tannery industry effluents. Similar results were reported by Qing et al. [53] in the steel industrial city (Anshan) of China. Significant levels of arsenic were found at site no. 6 (0.69 mg/L) due to motor vehicle repairs such as bodywork, chemicals in the cleaning and dismantling of vehicles, painting, soldering, hydraulic fluid, engine oil spills, leachates from used oils and greases, and spare parts that are frequently burnt on these sites. Similar results were investigated in a study by [54].

### 3.2. Isolation of Cultures and Their Minimum Inhibitory Concentration (MIC)

A total of 19 morphologically distinct fungi were retrieved from different soil samples. It can be assumed that metal-polluted habitats contain a wide range of fungi from all major taxonomic groups [55].

Fungal colonies were screened for heavy metal resistance in terms of MIC, and the highest tolerance/resistance (>5000 mg/L) was reported for isolates C1 and C3 for chromium and A2 and A6 for arsenic (Table 2). In a similar study which was conducted by Singh et al. [30], 54 fungal isolates were retrieved from arsenic-contaminated rice fields of middle Indo-Gangetic plains. Out of 54 isolates, 15 were tolerant to 10,000 mg L^−1^ of arsenates and were capable of bioaccumulation and volatilization. *Aspergillus oryzae* was the most prominent among them, with a bioaccumulation efficiency of 82%.

### 3.3. Morphological and Biochemical Characterization

**Figure 1:**
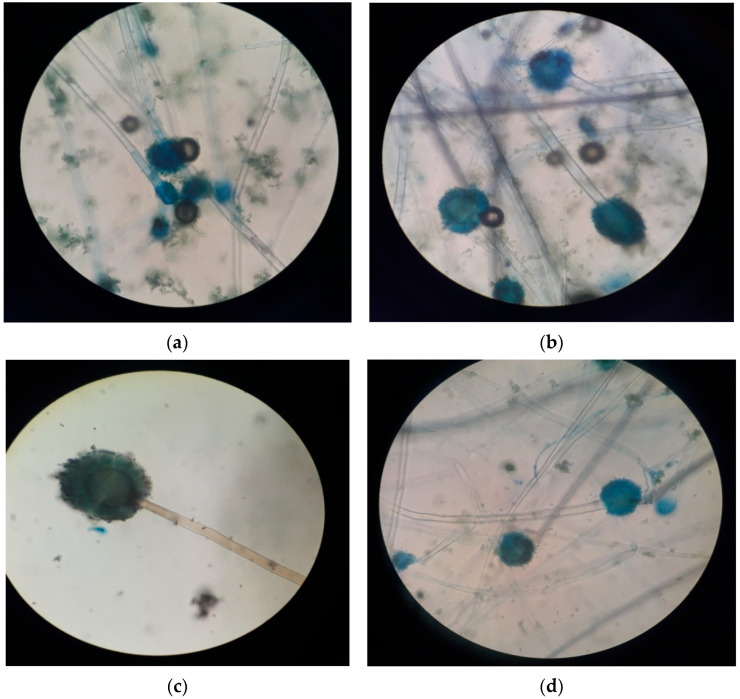
Microscopic images of (**a**) Isolate C1, (**b**) Isolate C3, (**c**) Isolate A2, and (**d**) Isolate A6.

The fungal isolates C1, C3, and A2, A6 were selected based on MIC and characterized morphologically and biochemically. The morphological characterization of selected fungal isolates is shown in Table 3, and microscopic images are shown in Figure 1. Enzyme bioassays and carbohydrate utilization patterns of selected fungal isolates are shown in Table 4. All the isolates were found to be positive for the carbohydrate assimilation and pectinase test. The fungal isolates C1 and A2 were found to be positive, and C3 and A6 were negative for the cellulase test. For the amylase test, the fungal isolate A6 was found to be positive, and the rest were negative. In the laccase test, the fungal isolates of C3 and A6 were found to be positive, and C1 and A2 were negative. A similar method of characterization was followed by Mohanty et al. [46]. The fungal isolates were identified as Aspergillus sp. on the basis of characterization.

### 3.4. Optimization of Cultural Conditions

**Figure 2:**
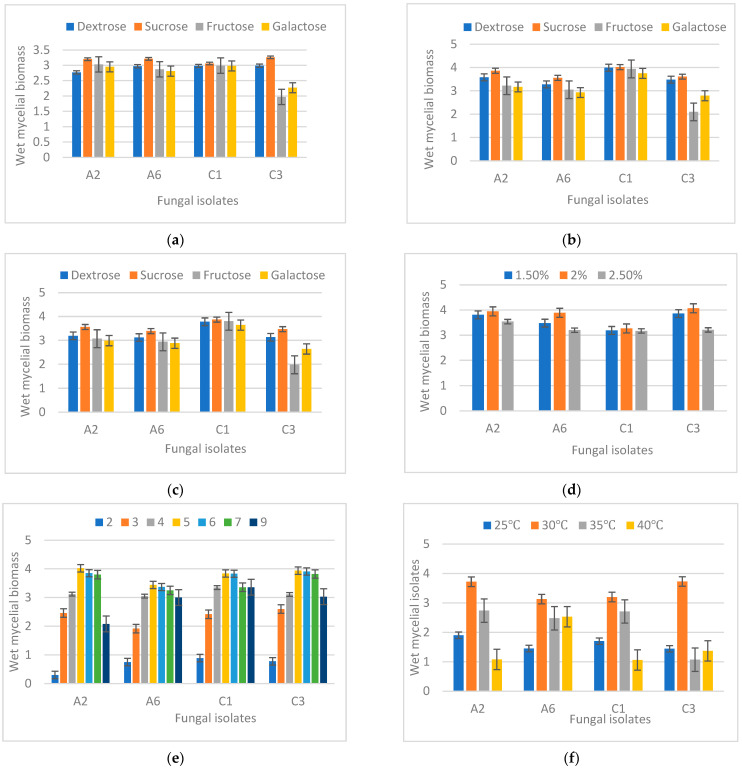
Effect of (**a**) Different carbon sources at 1% conc. (**b**) Different carbon sources at 2% (**c**) Different carbon sources at 3% (**d**) 1.5–2.5% of sucrose (**e**) pH 2.0–9.0 (**f**) The temperature of incubation 25 °C–40 °C on the growth of different fungal isolates.

Fungi require specific growth conditions. The fungal isolates C1, C3, and A2, A6 showed maximum growth when the media was supplemented with 2% (*w*/*v*) of sucrose. The wet weight of mycelial mass with 2% sucrose was 3.95 g, 3.89 g, 3.27 g, and 4.07 g for the isolates A2, A6, C1, and C3, respectively. In the temperature optimization studies, 30 °C reported the maximum fungal biomass production of 3.72 g, 3.13 g, 3.20 g and 3.73 g for the fungal isolates A2, A6, C1, and C3, respectively. Among the different pH of the medium, maximum growth was found at pH 5 by all the selected fungal isolates, whereas minimum growth was observed at pH 2. At the slightly acidic pH 5, the weight of the fungal pellet was 4.02 g, 3.44 g, 3.84 g, and 3.94 g for the fungal isolates A2, A6, C1, and C3, respectively (Figure 2).

### 3.5. Heavy Metals Removal Efficacy of Fungal Isolates

**Figure 3:**
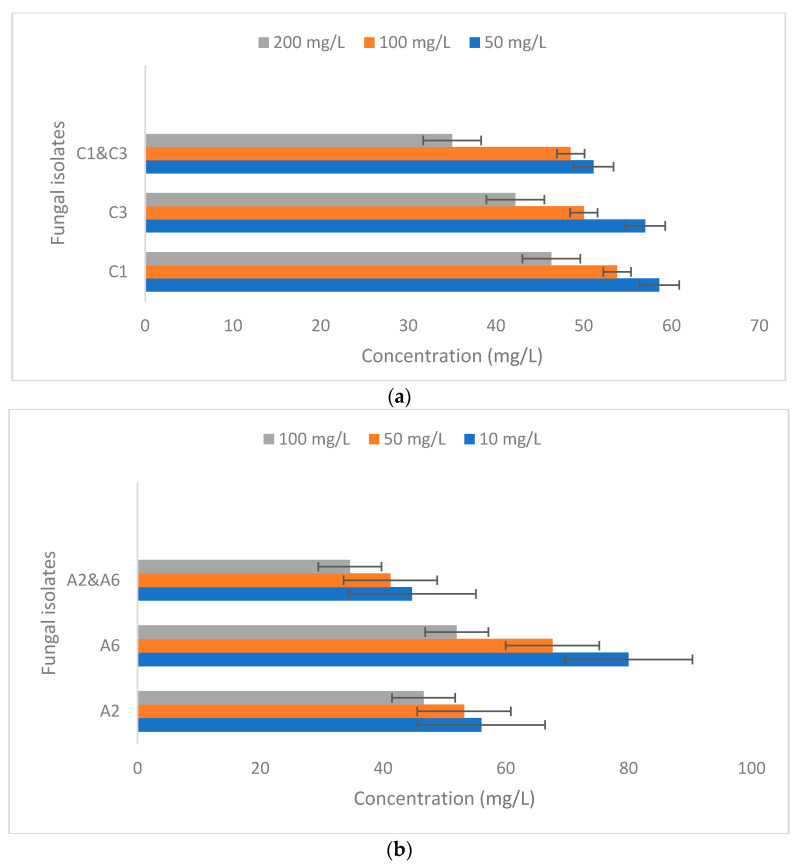
Removal of (**a**) Chromium by fungal isolates C1 and C3 and (**b**) Arsenic by isolates A2 and A6.

The fungal isolates C1 and C3 showed the highest removal of 58.60% and 57.00% at 50 mg/L chromium concentration, followed by 53.80% and 50% at 100 mg/L, and the minimum removal of 46.3% and 42.2% at 200 mg/L chromium concentration under the optimized conditions (Figure 3). In a similar study by Kumar and Dwivedi [35], for the bioremediation of chromium, the fungal isolate *Trichoderma lixii* CR 700 showed the removal of 99.4% at 50 mg/L of chromium concentration. For arsenic, the fungal isolates A2 and A6 showed the highest removal of 56.00% and 80.00% at ten mg/L arsenic concentration, followed by 53.20% and 67% at 50 mg/L, and the minimum removal of 46.6% and 52% were found at a 200 mg/L chromium concentration. In a similar study by Srivastava et al. [56], fungal isolates showed arsenic removal from 10.92% to 65.81% at ten mg/L of the arsenic concentration. In another study by Srivastava et al. [56], ten out of forty-five isolates were highly tolerant to arsenic. The isolates were able to remove 80% of arsenite and 85% of arsenate from the liquid medium. The variability might be due to different resistance tolerance mechanisms such as complexation, extracellular precipitation, crystallization, biosorption, the transformation of metals, efflux, etc., used by different fungal strains against metal [55].

### 3.6. Molecular Characterization of Potent Isolates

**Figure 4:**
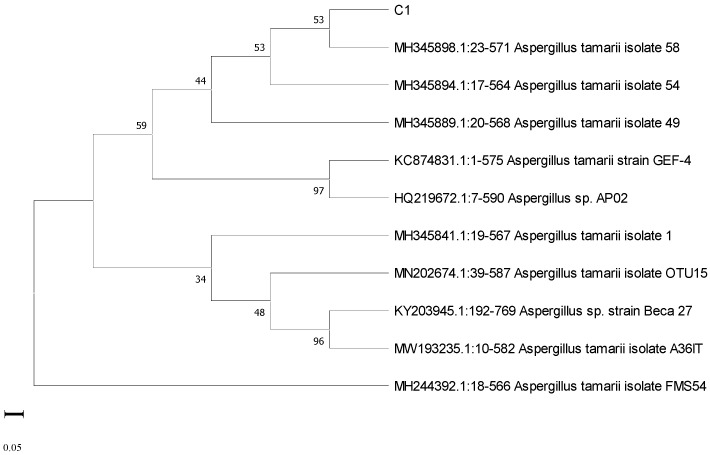
Phylogenetic tree of fungal isolate C1.

**Figure 5:**
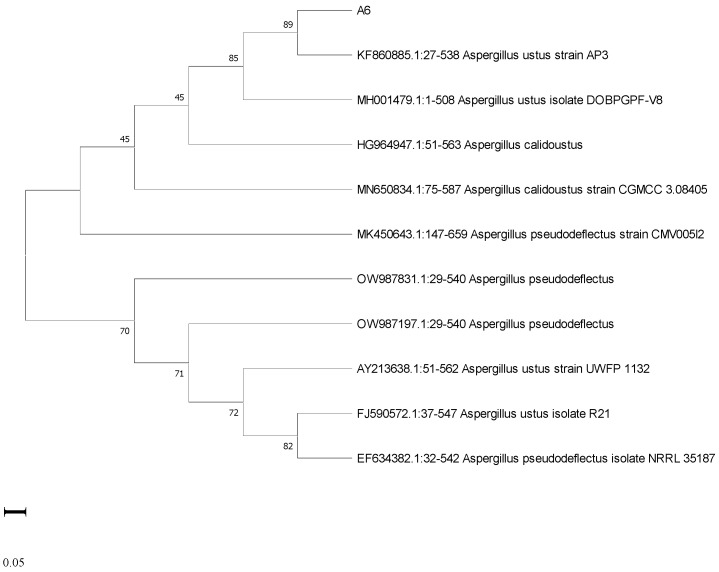
Phylogenetic tree of fungal isolate A6.

The phylogenetic trees of the fungal isolates C1 and A6 are shown in Figure 4 and Figure 5. ITS sequences were submitted in NCBI through GenBank (accession number OQ179906 for C1 and accession number OQ 179908 for A6). The fungal isolate C1 was identified as *Aspergillus tamarii,* and the isolate A6 was identified as *Aspergillus ustus*.

## 4. Conclusions

The current study reported the potential of fungal isolates for the removal of heavy metals, i.e., chromium and arsenic. The maximum removal was observed by up to 58.6% and 80% for chromium and arsenic, respectively. Fungal isolates could grow over a wide range of environmental conditions. The effective performance of fungal isolates in the present investigation provided a potent tool for the bioremediation of chromium and arsenic-contaminated sites. Further studies are required to check the metal (Cr & As) removal ability of isolates from actual waste waters to find a more viable alternative. Mycoremediation offers the ability to rehabilitate contaminated ecosystems affordably and successfully. Uncertainty arises from a lack of information regarding how different environmental elements can affect the rate and degree of biodegradation.

## Figures and Tables

**Table 1:** Physicochemical analysis of soil samples collected from various locations.

Site No.	Site (s)	Soil Texture/Type	pH	EC (mS/cm)	Total C (%)	Total N (%)	Total P (%)	Total K (%)	Heavy Metals (mg/L)	
									Cr	As
1	Automarket, Hisar Phase I	Loam	7.0	1.03	0.32	0.11	0.03	1.3	0.80	0.23
2	Jindal Steel Industry, Hisar	Silty clay loam	8.2	1.14	0.16	0.08	0.15	1.0	0.23	0.14
3	Ludas Outlet No. I	Loam	7.3	0.52	0.61	0.26	0.12	1.5	0.47	0.08
4	Auto-market, Hisar	Sand	7.1	1.18	0.21	0.07	0.06	1.1	1.53	0.10
5	Ludas Outlet No. II	Loam	8.4	0.66	0.68	0.31	0.28	2.1	0.22	0.20
6	Auto-market, Hisar	Sand	7.5	0.98	0.25	0.10	0.07	0.8	1.06	0.69
7	Farmer’s fields of village Dabra, Hisar	Sand	8.3	0.51	0.37	0.15	0.13	1.4	0.58	0.15
8	Farmer’s fields of village Kaimari, Hisar	Sandy loam	8.0	0.56	0.48	0.25	0.21	1.2	0.39	0.17
9	Farmer’s fields of village Shahpur, Hisar	Clay loam	8.2	0.60	0.49	0.22	0.24	1.5	0.45	0.18
10	Textile site of Panipat	Sandy	8.5	2.50	0.64	0.22	0.18	2.2	1.80	0.12
11	Tannery site of Panipat	Clay loam	8.8	1.45	0.59	0.26	0.13	2.7	2.23	0.11

**Table 2:** Fungal isolates and their tolerance toward heavy metals.

Metal(s)	Isolate (s)	MIC (mg/L)
Chromium	C1	>5000
	C2	800
	C3	>5000
	C4	800
	C5	2300
	C6	900
	C7	1200
	C8	900
	C9	1200
	C10	800
Arsenic	A1	400
	A2	>5000
	A3	2200
	A4	200
	A5	1400
	A6	>5000
	A7	100
	A8	400
	A9	200

**Table 3:** Morphological characterization of fungal isolates.

Fungal Isolate (s)	Morphological Characteristics				Microscopic Characteristics
	Colour	Margin	Elevation	Appearance	
C1	Olive green with white margin	Filliform	Raised	Cottony	Hyphae were hyaline, smooth, and septate, conidiophore unbranched, globular vesicle present and smooth globose spores were present
C3	Green	Entire	Flat	Powdery	Clear, smooth, septate hyphae, conidiospores present, uniseriate, spores globular in shape
A2	Olive green with white margin	Filliform	Raised	Cottony	Septate and hyaline hyphae, long unbranched conidiophores, globular vesicle
A6	Grey with white margin	Filliform	Raised	Cottony	Septate and smooth hyphae, long and unbranched conidiophores, uniseriate.

**Table 4:** Enzyme bioassays and carbohydrates utilization pattern.

Fungal Isolate (s)	Carbohydrate	Cellulase	Amylase	Laccase	Pectinase
C1	+	+	−	−	+
C3	+	−	−	+	+
A2	+	+	−	−	+
A6	+	−	+	+	+

+ = Positive; − = Negative.

## Data Availability

Not applicable.
